# Small-seeded *Hakea* species tolerate cotyledon loss better than large-seeded congeners

**DOI:** 10.1038/srep41520

**Published:** 2017-01-31

**Authors:** Sh-hoob El-Amhir, Byron B. Lamont, Tianhua He, George Yan

**Affiliations:** 1 Department of Environment and Agriculture, Curtin University, GPO Box U1987, Perth, WA 6845, Australia; 2 College of Horticulture and Forestry Sciences, Huazhong Agricultural University, Wuhan 430070, China

## Abstract

Six *Hakea* species varying greatly in seed size were selected for cotyledon damage experiments. The growth of seedlings with cotyledons partially or completely removed was monitored over 90 days. All seedlings perished by the fifth week when both cotyledons were removed irrespective of seed size. Partial removal of cotyledons caused a significant delay in the emergence of the first leaf, and reduction in root and shoot growth of the large-seeded species. The growth of seedlings of small-seeded species was less impacted by cotyledon damage. The rate of survival, root and shoot lengths and dry biomass of the seedlings were determined after 90 days. When seedlings were treated with balanced nutrient solutions following removal of the cotyledons, survival was 95–98%, but 0% when supplied with nutrient solutions lacking N or P or with water only. The addition of a balanced nutrient solution failed to restore complete growth of any species, but the rate of root elongation for the small-seeded species was maintained. Cotyledons provide nutrients to support early growth of *Hakea* seedlings, but other physiological roles for the cotyledons are also implicated. In conclusion, small-seeded *Hakea* species can tolerate cotyledons loss better than large-seeded species.

Cotyledons are essential for the early growth of seedlings until they become autotrophic^1^. During the first season, the roots of seedlings must reach soil containing sufficient moisture and nutrients to support growth, whereas the plumule must reach light to begin photosynthesis^2^. Additionally, reserved nutrients such as carbohydrates, organic nitrogen, phosphorus compounds, and inorganic ions are transported from the cotyledons into the developing seedling^3^,^4^,^5^. To investigate the role of cotyledons in early seedling development, we used species of the shrub genus, *Hakea*, as the model plant of our study.

*Hakea* (Proteaceae) is endemic to Australia. This genus is known for its wide range of seed sizes (2–500 mg) among the 150 extant species, 100 of which occur in south-western Australia, a region characterised by nutrient-impoverished soils and hot, dry summers^6^. Hakeas possess phanerocotylous epigeal seedlings (i.e., the cotyledons emerge from the seed coat, protrude above ground, spread laterally and are photosynthetically active) that is the most common type of seedling in the flora of south-western Australia^1^. Milberg & Lamont^1^ noted that early removal of cotyledons caused death of *Hakea* species and suggested that the cotyledons must have an important nutritional function in the early establishment of their seedlings. *Hakea* seedlings are unable to survive if both cotyledons are removed <7 days after germination. The primary reserves of nutrients that are present in the cotyledons of *Hakea* species include nitrogen (N), phosphorus (P), potassium (K), sodium (Na), magnesium (Mg), copper (Cu), calcium (Ca), sulphur (S), iron (Fe), manganese (Mn) and zinc (Zn). N and P are the most important nutrients supplied by the cotyledons to the young seedling^4^,^7^, whereas K, Ca and Mg are more likely obtained from the soil especially following fire^8^. P has an important functional role in energy transfer and metabolic regulation, whereas most N is used for the synthesis of enzymes that are necessary during seedling development, including those of photosynthetic cotyledons and the first leaves^9^. Other nutrients such as K, Mg and S have supportive roles for the growth and survival of seedlings that inhabit nutrient-impoverished soils^10^.

Lamont & Groom^4^ proposed that the role of cotyledons in seedling development could be replaced by the addition of mineral (rather than organic) nutrients to the soil. They found that the addition of N, P or (N + P) after cotyledon removal had limited benefit for the seedlings but that the addition of (P + N + K + Mg + S) restored the growth of the seedlings to those with intact cotyledons. It is not clear whether the seedlings rely only on N and P in the cotyledon reserve or they acquire some N and P from the soil at an early stage of growth.

The extent of dependence of seedlings on cotyledon nutrient reserves may be related to seed size and nutrient translocation efficiency. Groom & Lamont^11^ reported that the addition of a balanced nutrient solution to small-seeded species with intact cotyledons increased their growth 2.5-fold compared with those that received distilled water only. By contrast, the seedlings from large seeds have access to much larger resource reserves and rely on the reserves for much longer so that nutrient addition made little difference to their growth rate^12^. Large seeds may have an advantage under adverse environmental conditions, including nutrient-impoverished soils, as the seedlings are less reliant on soil nutrients for early growth^1^,^13^,^14^. Seedlings from large-seeded species generally survive and perform better than those from small-seeded species because the large-seeded species have extra nutrient reserve in the cotyledons to sustain root and shoot growth^15^,^16^. However, the loss of the cotyledons as a consequence of herbivory or physical forces (e.g., strong wind, frost) could have a severe effect on those species that rely on their cotyledons for nutrients to support early growth. Little is known about the association between seed size and cotyledon damage and the ability of seedlings to respond to various degrees of cotyledon damage.

In general, partial damage to seedlings from herbivory or other physical forces can be compensated for by regrowth. Seedlings can recover from the damage in several ways: for example, a seedling can recover from the lost leaf area by using stored assimilates to increase the photosynthetic rates of the remaining leaves, or can decrease the rate of leaf senescence, or can produce new leaves^17^. However, cotyledons cannot regrow when they are lost before the true leaves are produced, and damage to either or both cotyledons limits the ability of seedlings to develop root systems to access soil moisture and nutrient sources and prevents the accumulation of aboveground biomass from reaching a critical mass to ensure seedling survival^16^.

Despite the importance of cotyledons in the regeneration of plants^18^,^19^, some of the factors affecting seedling survival, including the interaction between seed size, seedlings and herbivores, are not completely clear^20^. It remains equivocal whether seedlings can survive and establish solely on the nutrients in the soil, particularly N and P, and whether the role of seed size is important in the acquisition of nutrients after the cotyledons are damaged. In south-western Australia, the greatest herbivory pressure experienced by *Hakea* seedlings comes from insects and small marsupials^21^. We hypothesise that seedlings from small seeds are better adapted to cotyledon damage than those of large seeds due to their less reliance on cotyledon reserves. Using Australian *Hakea* species as a model, the objectives of this study were to: 1) investigate the effects of the partial or complete removal of cotyledons on the early growth of seedlings to determine to what extent *Hakea* species tolerate cotyledon damage; 2) explore the role of seed (cotyledon) size in mitigating the negative impact of the partial or complete removal of the cotyledons on the early growth of seedlings; and 3) investigate whether the loss of cotyledons could be compensated for by the addition of critical nutrients to the soil, and if so whether such ability is seed-size dependent.

## Results

### The effects of cotyledon damage

Cotyledon removal significantly impacted seedling survival for all six *Hakea* species. All seedlings with intact or partially removed cotyledons survived when treated with water for 30 days, whereas 100% mortality was recorded for seedlings with their cotyledons completely removed and receiving water only. Most deaths occurred within 4–10 days after removal of the cotyledons. The surviving seedlings showed that medium to severe cotyledon damage (50 and 75% removal) significantly delayed the emergence of the first true leaves compared with that of Con+ (Table 1). For *H. francisiana,* and *H. petiolaris,* the two species with the smallest seeds, the first true leaf emerged 4–8 days after the cotyledons expanded in seedlings with intact cotyledons, whereas for the other four species with large seeds, the first true leaf emerged 12–22 days after the cotyledons expanded. Longer delays were observed for seedlings with higher proportions of cotyledon removal and seedlings from species with larger seeds (Tables 1 and 2). The longest delays (11 days) were recorded for seedlings of *H. platysperma,* the species with the largest seeds among the six species studied.

**Figure 1:**
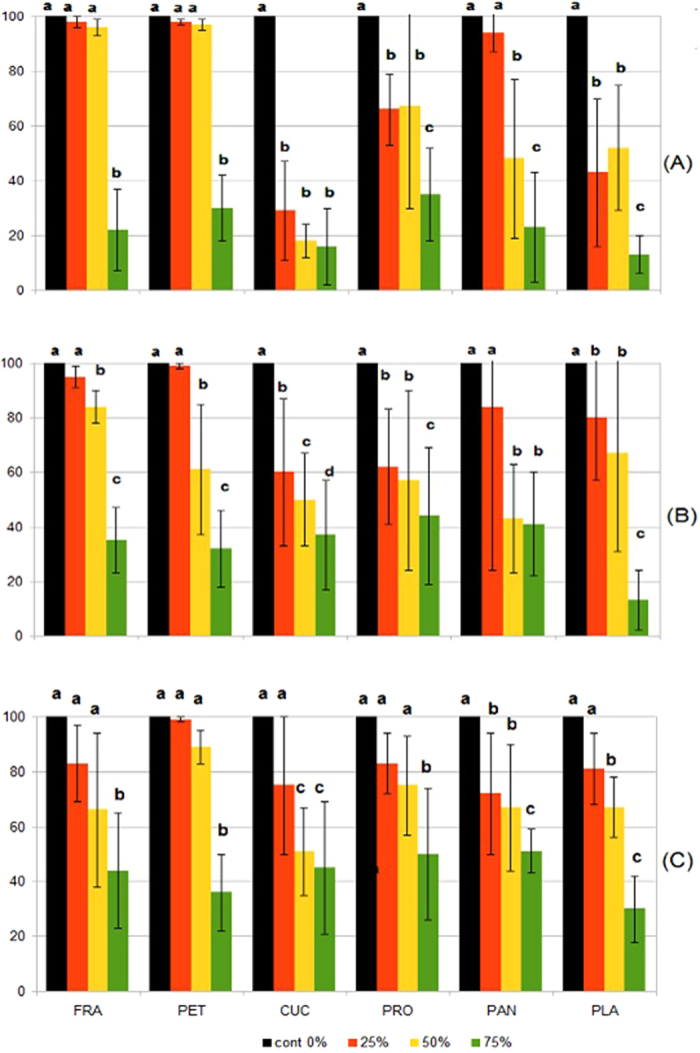
Root and shoot dry mass (**A**,**B**) and root length (**C**) of 90-day old seedlings of six *Hakea* species with nil or partial removal of cotyledons. Measurements were converted to % using values of the controls as the basis (100%) to facilitate comparisons between treatments. Different letters above bars indicate a significant difference between treatments at *P* < 0.05. Treatment: cont 0%: control with 0% cotyledon removal; 25%: 25% cotyledon removal, etc. Plant species: FRA, *H. francisiana;* PET, *H. petiolaris*; CUC, *H. cucullata;* PRO, *H. prostrata*; PAN, *H. pandanicarp*; and PLA, *H. platysperma*.

To study the impact of cotyledon removal on seedling growth, three sets of measurements (at 30, 60 and 90 days) for root and shoot length and dry biomass were taken. Examination of the data revealed that trends were consistent among treatments for the three sets of measurements. Only data from the 90-day measurements are analysed and presented here. Tolerance to partial removal of cotyledon varied among the six species. The variation in tolerance seemed to be related to the seed size of the species studied. When 25 to 50% of cotyledons were removed, root length and root dry biomass were not significantly different from those of seedlings with cotyledons intact for *H. francisiana* and *H. petiolar* is, the two species with the smallest seeds among the six species studied (Fig. 1). In comparison, the four large-seeded species were much less tolerant to cotyledon removal, showing marked reductions in seedling growth when cotyledons were partially removed. For all species, growth of seedlings was severely impacted by 75% removal of cotyledon irrespective of seed size (Fig. 1).

### Nutrient compensation to cotyledon damage

For all six species studied, seedlings with intact cotyledons survived when treated with water for 90 days, whereas 100% mortality was recorded for seedlings with their cotyledons removed and receiving water only. By contrast, when supplemented with the (N + P + K) solutions, 95–98% of seedlings survived for more than 30 days with the cotyledons removed. For all species, seedlings with the cotyledons removed that received the (P + K) or (N + K) nutrient solutions did not survive. In comparison, 70–90% of seedlings of *H. prostrata, H. pandanicarpa* and *H. platysperma,* the three large-seeded species, survived with the (N + P) solution, but none of the seedlings of the three small-seeded species survived when receiving the (N + P) solution treatment.

As with the cotyledon removal experiment, seedlings treated with nutrient supplement solutions also showed significant delays in the emergence of the first true leaves (Table 3). Compared with seedlings having intact cotyledons, there was an average of 8–12 days of delay when supplemented with the (N + P + K) nutrient solutions, and the delay increased to 29 to 41 days when supplemented with (N + P) solutions for the surviving seedlings of the three large-seeded species. When expressed in relative terms, the delay in the emergence of the first true leaves ranges from 220% to 33% when treated with the (N + P + K) nutrient solutions. The two small-seeded species, *H. francisiana* and *H. petiolaris*, showed the highest percentage of delays in comparison to their controls (Table 3).

With the cotyledons removed, seedlings that were treated with nutrient supplement solutions grew significantly slower and accumulated less biomass than those with intact cotyledons (Table 4). The trend is consistent across the six species. The percentage reduction in total biomass ranges from 45 to 68%. These reductions increased to 79–87% when potassium is omitted from the supplementary solutions (Table 4). However, when root mass was examined separately, a different pattern emerges among the six species. For *H. francisiana* and *H. petiolaris,* the two species with the smallest seeds, there was no significant reduction in root mass when treated with N + P + K solutions following cotyledon removal. By contrast, there were significant reductions in root mass for the four large-seeded species under the same nutrient treatment (Table 4).

Following the removal of the cotyledons, root lengths of seedlings treated with the (N + P + K) solutions were unaffected for *H. francisiana, H. petiolaris,* and *H. cucullata,* the three smallest-seeded species. In comparison, the three large-seeded species showed significant reductions in root length when treated with the (N + P + K) and (N + P) solutions. There was an average of 50% reduction across the three species. The reduction was even greater when potassium was omitted from the treatment solutions (Table 4). MANOVA tests indicated that seed size significantly interacted with nutrient supply after cotyledon removal (*P* < 0.01).

The root: shoot mass ratios of the six species are presented in Table 4. The ratio varied from 1.4 to 2.0 for seedlings with cotyledon intact across the six species. With the exception of the large-seeded *H. platysperma*, all species treated with the (N + P + K) or (N + P) solutions (if they survived after cotyledon removal) maintained their root: shoot mass ratios.

## Discussion

### Seed size and tolerance to cotyledon damage

Partial or complete removal of the cotyledons negatively impacted early growth of the seedlings of the six *Hakea* species examined. The extent of impact depended on the severity of damage, with death resulting among all species when cotyledons were completely removed. Severe damage significantly delayed the emergence of true leaves and reduced growth of root and shoots in all six species, indicating that the seedlings were unlikely to survive the first summer drought after germination. Seed size appears to affect the reaction to cotyledon damage, with the four larger-seeded species showing greater sensitivity to cotyledon removal than the two smaller-seeded species (Tables 1 and 3). The largest-seeded species, *H. platysperma*, was particularly adversely affected by the 75% cotyledon removal treatment. Nevertheless, the presence of cotyledons is just as important for early seedling growth for the small-seeded species as for the large-seeded species in nutrient-impoverished soils.

These results are consistent with the majority of previous studies, showing that damage to cotyledons has indirect effects on fitness through reducing the rate of plant growth. For many plant species, damage to cotyledons can negatively influence early seedling growth, limiting the ability of the seedling to develop a root system to access soil moisture and nutrients and preventing the above ground biomass from reaching a critical size to ensure self-sustainability^1^,^22^,^23^. For example, Hocking & Steer^24^ reported that cotyledon removal resulted in reduced growth and leaf numbers per plant during the early growth of oilseed and sunflower seedlings. Similarly, large negative effects of cotyledon removal on the biomass and seedling survival were reported for *Quercus robur*^25^ and *Q. mongolica*^19^.

It is interesting to note that it took less cotyledon damage to severely impact growth of the larger-seeded species. Larger seeds produce much faster growing seedlings that rely more heavily on their cotyledons for rapid growth. Even with 50% cotyledon removal, biomass of the largest-seeded species, *H. platysperma*, was ten times that of the smallest-seeded species, *H. francisiana*. In the absence of external nutrients in the growing medium it was not possible for the nutritional role of the cotyledons to be supplemented from the soil. This has major implications for survival of the larger-seeded *Hakea* species, for their survival relies on the ability of their sinker roots to keep pace with the declining water availability at the soil surface and thus avoid drought^1^,^15^.

The findings that seedlings of small-seeded *Hakea* species are more resistant to cotyledon damage are contrary to the common belief concerning a positive relationship between seed size and seedling recovery from cotyledon damage^12^,^22^,^26^,^27^. There are two possible explanations for the greater tolerance of seedlings to minor cotyledon damage in small-seeded species. First, leaf emergence and development was more rapid in seedlings from small-seeded species than those from large-seeded ones. As observed in our study, the average time from cotyledon emergence to the expansion of the first true leaf was 8 days for small-seeded *H. francisiana,* and 20 days for large-seeded *H. platysperma.* Thus, the smaller-seeded species may have already made greater use of the cotyledons before they were removed. Second, seedlings of small seeds are also more reliant on the soil nutrients for early seedling growth than seedlings of large seeds^1^,^4^, they may be more efficient in absorbing and translocating soil nutrients at an early stage of seedling development. Seedlings derived from large seeds, on the other hand, being more reliant on nutrient reserves stored in the cotyledons, may be slow in developing their capacity to absorb and translocate nutrients from the soil.

The quick germination and early emergence of true leaves, together with unimpaired development of the root system under light-medium cotyledon damage, place seedlings of small seeds at a selective advantage compared with seedlings of large seeds. These effects enhance the chance of successful seedling establishment by twofold. First, unimpaired root development ensure roots quickly reach soil moisture and nutrients, and the early emergence of true leaves enable seedlings to become autotrophic sooner. Second, the short seed germination time reduces the exposure to adverse conditions such as high herbivory pressure when they are most vulnerable.

### The effects of nutrient supplements on seedling development

Our results indicate that the role of cotyledons could be partially substituted by the addition of essential nutrient elements. Survival of seedlings approached 100% when a balanced nutrient solution was supplied after removal of the cotyledons (and 0% when they were not). However, growth of the seedlings could not be compensated for completely by the addition of a balanced nutrient solution. These results confirm the findings of Lamont & Groom^4^, working on other *Hakea* species (with seed mass intermediate to those studied here). In their study, they showed that nutrient solutions could be devised to compensate for the effect of cotyledon loss to a large extent. Cotyledons contribute to the growth and development of seedlings by acting as the nutrient reserve and supplying chemical resources such as hormones and carbohydrates from the reserve and/or through photosynthetic activities^28^,^29^. Kitajima^28^ demonstrated that the loss of photosynthetic capacity in the cotyledons could have a greater effect on seedlings than the loss of the nutrients that are stored in the cotyledons. It appears that supplementary solutions containing nitrogen, phosphorus and potassium after total loss of the cotyledons were not able to replace the photosynthetic function of the cotyledons.

The findings that growth of the root system, measured as root length and mass, continues even after cotyledon removal in the two small-seeded species is intriguing. This suggests that root growth of small-seeded species is maintained in an effort to reach water (indirect response) or nutrients (direct response) even after damage to the cotyledons. Small-seeded species rely on soil sources of nutrients much earlier than large-seeded species due to their small internal nutrient reserves^1^,^4^. In water-limited habitats, such as in south-western Australia, the rapid elongation of the taproot increases the chances of maintaining contact with soil water and hence the survival of the seedling^1^,^30^.

Young plants of woody shrub species raised in pots under low nutrient conditions typically have a root: shoot ratio on a mass basis of ~0.43 reducing to ~0.30 in the presence of nutrient supplements^31^. The high root: shoot ratio (1.4–2.0) at 90 days for the six *Hakea* species studied here can be attributed to two factors: the complete absence of soil nutrients and the deep (100 cm long) pots we used. This design simulates well the field conditions in south-western Australia, a region characterised by nutrient-impoverished soils of great depth, and shows how root growth may be promoted when soil volume is not limiting as usually occurs in pot experiments.

Our results show that cotyledons are an essential source of N and P for 90-day-old seedlings but not always of K. All decotyledoned plants died when supplied a balanced nutrient solution lacking P or N, whereas the four larger-seeded species survived in a solution lacking K, though none grew anywhere near as well as seedlings with intact cotyledons (Table 3, Fig 1). It is worth noting that there was a minor amount of K (2% of full) in the (N + P) treatment (from KH_2_PO_4_). Poot & Lambers^32^ suggested that a K concentration of 200 μM is required for normal growth in *Hakea.* The presence of 4 μM of K in the (N + P) solution would have a negligible effect on growth, as evident by total death of the small-seeded species in that treatment. It is possible that K is transported earlier than P or N into the seedling as it is in ionic form rather than bound organically. K therefore may be mobilized first so that it is not as critical by the time cotyledons were removed or K is not as essential as P and N for early growth. Both interpretations are plausible as, for two of the large-seeded species, the biomass and root length were not different between the control and the (N + P) treatments. This provides support for the contention of Stock *et al*.^8^ that K is usually supplied (later) from post-fire ash when seedling recruitment is most likely.

Although the survival of decotyledoned seedlings was assisted by access to additional nutrients in the soil, the growth of seedlings was significantly retarded (Tables 3 and 4, Fig. 1). The growth of seedlings was severely delayed when the cotyledons were removed. This delay would place the seedling at a disadvantage when stressed by factors such as competition. Following the loss of cotyledons, even with the additional supply of all essential nutrients, there were significant reductions in biomass and a long delay in the emergence of the first true leaves. It remains unclear whether this was because the nutrient solution supplied did not meet the specific requirements of the species we used. The content of P in our nutrient solution was low compared with that of the soil^7^. Alternatively, other essential functions of the cotyledons (e.g. providing organic compounds) were now prevented. The small-seeded species were better able to tolerate the loss of cotyledons by maintaining root growth but only when they had access to soil nutrients (Table 1). By contrast, the species with large seeds were more severely affected by the loss of the cotyledons as they depend on the nutrients in the cotyledons for survival and early growth and make little use of soil nutrients, especially N and P, at the early stages of growth^4^. Thus, large-seeded *Hakea* species will be more sensitive to herbivory or other causes of cotyledon loss than small-seeded species.

Our findings are consistent with earlier studies that seedlings from small seeds are dependent on external resources soon after their germination^1^,^4^,^33^. In contrast, seedlings of *Quercus mongolica* with large acorns rely on the cotyledon for an extended period of time^34^. The contention that seedlings from small seeds are better adapted to damage of cotyledons than those of large seeds due to their less reliant on the cotyledon reserves and shortened germination and leaf emergence times are supported by the findings of the present study.

## Materials and Methods

Six *Hakea* species were selected for the present study (Table 2). These species were chosen for their diverse phylogenetic backgrounds and a wide spectrum of seed sizes that ranged from 8 mg (*H. francisiana*) to 500 mg (*H. platysperma*). Seeds were sourced from a commercial seed supplier (Nindethana Seed Services, Albany, Western Australia). Seeds were germinated in filter-paper lined petri dishes in environmentally controlled germination cabinets at 15 °C with a 12–12 hour light-dark cycle (though light is not required for their germination). The germinants were transplanted into pots constructed of PVC irrigation pipe (100 cm tall, 5 cm diameter) to encourage root growth. The pots were filled with white silica sand that had been washed with deionised water. All germinants were planted within the top 1 cm of the sand.

The experiments were conducted in a glasshouse on the Bentley campus of Curtin University from 2 July to 25 November 2013. The germinants were grown until the cotyledons expanded. It took 4 to 7 days after germination for the cotyledon to flatten. Cotyledon manipulations were initiated immediately after. The time of cotyledon removal is important in determining whether the seedling will survive. The removal of cotyledons later than seven days was less effective because most of the essential substances had already been transported from the cotyledons to the remainder of the plant or the true leaves had emerged and further growth was independent of the cotyledons, while early removal of the cotyledons can cause death from the wound impact^4^.

The relative importance of the cotyledons for seeding growth and survival was evaluated using two experiments. In the first, the manipulation treatments involved either 1) 0% cotyledon removal (control), 2) 25% of each cotyledon removed, 3) 50% of each cotyledon removed, 4) 75% of each cotyledon removed, or 5) 100% of both cotyledons removed. The cotyledons were carefully excised using a sterilised razor blade. All seedlings were manually treated with deionised water every three days to ensure sufficient soil moisture, while no additional nutrients were introduced.

The emergence of the true leaves was monitored for all treatments. Every 30 days after cotyledon manipulation, five seedlings from each treatment, including the control, in each of the six species were randomly selected and harvested. During harvest, root length (length from ground level to the tip of the longest root) was measured as an extensive root system is a key trait in adaptation to southwest Australia’s drought-prone environment^16^. The plants were then washed free of sand particles before oven-drying at 60 °C for 48 h, and the biomass of the roots and shoots were measured.

In the second experiment, 50 seedlings of each species had their cotyledons removed (both cotyledons were sliced off with a razor blade at the nodes) with an equal number left intact as controls. These seedlings were subjected to nutrient supplementation treatments. The full solutions contained the following compounds: Ca(NO_3_)_2_, K_2_SO_4_, KH_2_PO_4_, MgSO_4_, MnSO_4_, ZnSO_4_, CuSO_4_, H_3_BO_3_, Na_2_MoO_4_, and Fe-EDTA. Four variants of the nutrient solutions were prepared as follows:

1. Full (balanced) nutrient solution (N + P + K): with all the above nutrients included;
2. Phosphorus and potassium solution (P + K): the full nutrient solution minus Ca(NO_3_)_2_;
3. Nitrogen and potassium solution (N + K): the full nutrient solution minus KH_2_PO_4_;
4. Nitrogen and phosphorus solution (N + P): the full nutrient solution minus K_2_SO_4_.

Note that Ca was also omitted from the (P + K) treatment while 1.96% of K used in the Full solution remained in the (N + P) treatment. All solutions were prepared as a 10× stock that was then diluted with deionised water before being applied to the seedlings. All seedlings were watered with deionised water before the cotyledons were removed for further treatment. In each treatment, individual seedlings were watered once a week with 50 mL of a diluted nutrient solution or the identical amount of deionised water. Details of the nutrient supplementation experiment and composition of the nutrient solutions are presented in Table 5.

The growth and survival of the seedlings were monitored following treatment. Seedlings that turned dry and brown and that had no new growth for 10 days were recorded as “dead”. Every 30 days from emergence, five surviving seedlings were harvested per treatment. At harvest, the entire plant was removed from its container, and the roots and shoots were washed clean. Any residual cotyledons were removed from those seedlings in Con+ to facilitate the comparison of growth with those seedlings with cotyledons removed at the start of treatment. The plants were separated into roots and shoots. For each plant, root length and shoot and root mass were measured and recorded. The small-seeded species emerged early, whereas the large-seeded species required more than a week before the emergence of their cotyledons. The date of the harvests varied depending on the emergence time of the cotyledons, so that growth time was consistent across all species and treatments. Harvest time between different species varied by a few days (1–3 days). As all seedlings were grown in glasshouse, variations in growth conditions during this short period of time would be moderated to some extent. Impact on seedling growth by this variation in growth conditions was minimal if any.

### Data analysis

Linear mixed effects were used to test for differences in leaf emergence, among the control, and partial or total removal of the cotyledons (0, 25, 50, 75 and 100%). Size and biomass of seedling parts harvested after 90 days of growth were analysed by ANOVA to test for differences between the control (Con+) and full nutrient (N + P + K) treatments, and full nutrients with key elements omitted [(N + P) only, as 100% mortality was recorded for the Con−, (P + K) and (N + K) treatments]. MANOVA were used to examine the relationship of seed size on tolerance of the removal of the cotyledons and whether nutrients compensated the growth of seedlings (total biomass, root length, ratio of root: shoot biomass). All statistical analyses were implemented using SPSS (SPSS Inc., Chicago, Illinois) with significance accepted at *P* < 0.05.

## Additional Information

**How to cite this article**: El-Amhir, S.-h. *et al*. Small-seeded *Hakea* species tolerate cotyledon loss better than large-seeded congeners. *Sci. Rep.* **7**, 41520; doi: 10.1038/srep41520 (2017).

**Publisher's note:** Springer Nature remains neutral with regard to jurisdictional claims in published maps and institutional affiliations.

## Figures and Tables

**Table 1:** Time (days) to emergence of the first leaf (±SE) following various levels of cotyledon removal.

Species	Control	25%	50%	75%
*H. francisiana*	8 ± 1	8 ± 1 ns	11 ± 1*	12 ± 1**
*H. petiolaris*	4 ± 1	4 ± 1 ns	6 ± 1*	8 ± 1**
*H. cucullata*	12 ± 1	16 ± 1*	16 ± 1*	20 ± 2**
*H. prostrata*	12 ± 1	17 ± 2*	17 ± 1*	20 ± 2**
*H. pandanicarpa*	22 ± 2	25 ± 2*	27 ± 2*	28 ± 3**
*H. platysperma*	20 ± 2	22 ± 3 ns	25 ± 3*	28 ± 3**

Control: cotyledons intact; 25%: 25% of cotyledon area removed, etc. Seedlings with both cotyledons completely removed (100%) died within 30 days before harvest so these results are omitted. Ns: not significant; *P < 0.05; **P < 0.01.

**Table 2:** Average seed mass (±SD) and cotyledon area (±SD) after germination, and the informal taxonomic group to which the species belongs, of the six *Hakea* species used in the study.

Species	Seed mass (mg)#	Cotyledon area (mm^2^)#	Taxonomic group##
*H. francisiana*	8 ± 2	75 ± 8	Strumosa
*H. petiolaris*	16 ± 2	88 ± 10	Petiolaris
*H. cucullata*	29 ± 3	102 ± 12	Cucullata
*H. prostrata*	60 ± 7	117 ± 13	Prostrata
*H. pandanicarpa*	100 ± 12	232 ± 23	Ceratophylla
*H. platysperma*	501 ± 60	598 ± 24	Platysperma

^#^Data from Groom & Lamont^6^ and Kew Gardens^35^. ^##^Adapted from Barker *et al*.^36^.

**Table 3:** Time ± SE in days to leaf emergence of six *Hakea* species receiving nutrient supplements following the removal of cotyledons (days were calculated using the emergence of the cotyledons as day 0).

Species	Treatment						
	Con+	(N + P)	%	(N + P + K)	%	P values	
	Days ± SE	Days ± SE		Days ± SE		Con+ vs (N + P)	Con+ vs (N + P + K)
*H. francisiana*	8 ± 1	—		20 ± 4	150	—	<0.001
*H. petiolaris*	5 ± 1	—		16 ± 4	220	—	<0.001
*H. cucullata*	12 ± 1	—		20 ± 3	67	—	<0.001
*H. prostrata*	14 ± 1	43 ± 5	207	22 ± 4	57	<0.001	<0.01
*H. pandanicarpa*	24 ± 2	65 ± 4	171	32 ± 5	33	<0.001	<0.05
*H. platysperma*	22 ± 2	62 ± 6	182	34 ± 6	55	<0.001	<0.01

Percentages are treatment delay ratio relative to the control and are calculated as: (days of leaf emergence − days of leaf emergence of control)/days of leaf emergence of control. Cont+: control, seedlings with cotyledon intact; (N + P + K): solution contains nitrogen, phosphorus and potassium; (N + P): solution contains nitrogen and phosphorus. For details of the solution composition, refer to Table 5.

**Table 4:** Tolerance to the removal of cotyledons when supplemented with nutrients of six *Hakea* species.

	Con+	(N + P)	(N + P + K)	(N + P)		(N + P + K)	
				*F*	*P*	*F*	*P*
**Total biomass (mg)**							
*H. francisiana*	31 ± 6	—	18 ± 2	—	—	7.2	*
*H. petiolaris*	138 ± 18	—	47 ± 4	—	—	13.3	**
*H. cucullata*	70.8 ± 17	—	23 ± 4	—	—	10.3	**
*H. prostrata*	170 ± 16	36 ± 5	71 ± 6	8.9	**	4.5	*
*H. pandanicarpa*	120 ± 30	26 ± 6	50 ± 8	10.9	**	5.5	*
*H. platysperma*	577 ± 192	75 ± 36	189 ± 72	14.0	**	7.9	**
**Root length (cm)**							
*H. francisiana*	12.6 ± 1.6	—	11.0 ± 1.8	—	—	0.4	ns
*H. petiolaris*	30.9 ± 6.9	—	27.1 ± 1.9	—	—	0.6	ns
*H. cucullata*	20.2 ± 2.3	—	16.6 ± 0.9	—	—	0.5	ns
*H. prostrata*	34.2 ± 5.1	17.5 ± 1.7	18.0 ± 3.1	4.8	*	4.9	*
*H. pandanicarpa*	29.3 ± 6.1	8.9 ± 2.8	14.8 ± 6.1	15.2	**	7.2	*
*H. platysperma*	42.6 ± 7.1	17.9 ± 2.1	27.9 ± 1.4	25.6	**	7.4	*
**Root mass (mg)**							
*H. francisiana*	17.8 ± 4.2	—	13.6 ± 1.7	—	—	1.6	ns
*H. petiolaris*	40.0 ± 13.2	—	23.4 ± 4.7	—	—	1.1	ns
*H. cucullata*	35.7 ± 8	—	12.4 ± 2.4	—	—	9.0	**
*H. prostrata*	67.5 ± 5.2	15.9 ± 4.2	25.9 ± 3.0	7.9	**	5.0	*
*H. pandanicarpa*	72.3 ± 15	14.7 ± 3.0	27.5 ± 5.2	13.7	**	6.9	*
*H. platysperma*	281 ± 82	48.1 ± 19.1	131.7 ± 54.1	20.8	**	7.0	*
**Root: shoot mass ratio**							
*H. francisiana*	1.4 ± 0.2	—	1.2 ± 0.1	—	—	3.5	ns
*H. petiolaris*	1.5 ± 0.2	—	1.0 ± 0.1	—	—	0.2	ns
*H. cucullata*	1.2 ± 0.2	—	1.2 ± 0.1	—	—	0.0	ns
*H. prostrata*	0.7 ± 0.2	0.8 ± 0.0	0.6 ± 0.0	0.7	ns	1.0	ns
*H. pandanicarpa*	1.7 ± 0.2	1.8 ± 0.1	1.3 ± 0.2	0.0	ns	2.1	ns
*H. platysperma*	1.9 ± 0.2	1.1 ± 0.1	1.3 ± 0.1	7.9	**	7.7	*

MANOVA were carried out using a 2-tailed, mixed-effect model for seedling growth of the six *Hakea* species. Cont +: seedlings with cotyledon intact, (N + P + K): solutions contain nitrogen, phosphorus, and potassium; (N + P): solutions contain nitrogen and phosphorus. For details of the solution composition, refer to Table 5. All measurements were for 90-day-old seedlings. Total biomass is biomass of roots and shoots combined. Where significant, the control exceeded the treatment. *P < 0.05; **P < 0.001; ns, not significant (P > 0.05). -No seedlings survived the treatment.

**Table 5:** Design of the nutrient supplementation experiment and the composition of nutrient solutions.

Codes	Nutrient solutions concentration (μM)										
	NO_3_	H_2_PO_4_	K_2_SO_4_	Ca(NO_3_)_2_	MgSO_4_	MnSO_4_	ZnSO_4_	CuSO_4_	Na_2_MoO_4_	Fe-EDTA	H_3_BO_3_
Con+	0	0	0	0	54	0.24	0.10	0.018	0.030	40	2.4
Con−	0	0	0	0	54	0.24	0.10	0.018	0.030	40	2.4
(N + P + K)	400	4	204	200	54	0.24	0.10	0.018	0.030	40	2.4
(P + K)	0	4	204	0	54	0.24	0.10	0.018	0.030	40	2.4
(N + K)	400	0	204	200	54	0.24	0.10	0.018	0.030	40	2.4
(N + P)	400	4	4	200	54	0.24	0.10	0.018	0.030	40	2.4

Con+: Cotyledons intact; Con−: Cotyledons removed.
